# Cytological and Gene Profile Expression Analysis Reveals Modification in Metabolic Pathways and Catalytic Activities Induce Resistance in *Botrytis cinerea* Against Iprodione Isolated From Tomato

**DOI:** 10.3390/ijms21144865

**Published:** 2020-07-09

**Authors:** Ambreen Maqsood, Chaorong Wu, Sunny Ahmar, Haiyan Wu

**Affiliations:** 1 Guangxi Key Laboratory of Agric-Environment and Agric-Products Safety, Agricultural College of Guangxi University, Nanning 530004, Guangxi, China; ambreenagrarian@gmail.com (A.M.); nongyekejian@163.com (C.W.); 2 Department of Plant Pathology, University College of Agriculture and Environmental Sciences, The Islamia University of Bahawalpur, Bahawalpur 63100, Pakistan; 3 National Key Laboratory of Crop Improvement Genetics, College of Plant Sciences and Technology, Huazhong Agricultural University, Wuhan 430070, Hubei, China; sunny.ahmar@yahoo.com

**Keywords:** *Botrytis cinerea*, tomato, iprodione, mutant, transcriptome analysis, metabolism, catalytic activity

## Abstract

Grey mold is one of the most serious and catastrophic diseases, causing significant yield losses in fruits and vegetables worldwide. Iprodione is a broad spectrum agrochemical used as a foliar application as well as a seed protectant against many fungal and nematode diseases of fruits and vegetables from the last thirty years. The extensive use of agrochemicals produces resistance in plant pathogens and is the most devastating issue in food and agriculture. However, the molecular mechanism (whole transcriptomic analysis) of a resistant mutant of *B. cinerea* against iprodione is still unknown. In the present study, mycelial growth, sporulation, virulence, osmotic potential, cell membrane permeability, enzymatic activity, and whole transcriptomic analysis of UV (ultraviolet) mutagenic mutant and its wild type were performed to compare the fitness. The EC_50_ (half maximal effective concentration that inhibits the growth of mycelium) value of iprodione for 112 isolates of *B. cinerea* ranged from 0.07 to 0.87 µg/mL with an average (0.47 µg/mL) collected from tomato field of Guangxi Province China. Results also revealed that, among iprodione sensitive strains, only B67 strain induced two mutants, M0 and M1 after UV application. The EC_50_ of these induced mutants were 1025.74 μg/mL and 674.48 μg/mL, respectively, as compared to its wild type 1.12 μg/mL. Furthermore, mutant M0 showed higher mycelial growth sclerotia formation, virulence, and enzymatic activity than wild type W0 and M1 on potato dextrose agar (PDA) medium. The *bctubA* gene in the mutant M0 replaced TTC and GAT codon at position 593 and 599 by TTA and GAA, resulting in replacement of phenyl alanine into leucine (transversion C/A) and aspartic acid into glutamic acid (transversion T/C) respectively. In contrast, in *bctubB* gene, GAT codon at position 646 is replaced by AAT and aspartic acid converted into asparagine (transition G/A). RNA sequencing of the mutant and its wild type was performed without (M0, W0) and with iprodione treatment (M-ipro, W-ipro). The differential gene expression (DEG) identified 720 unigenes in mutant M-ipro than W-ipro after iprodione treatment (FDR ≤ 0.05 and log2FC ≥ 1). Seven DEGs were randomly selected for quantitative real time polymerase chain reaction to validate the RNA sequencing genes expression (log fold 2 value). The gene ontology (GO) enrichment and Kyoto encyclopedia genes and genomes (KEGG) pathway functional analyses indicated that DEG’s mainly associated with lysophopholipase, carbohydrate metabolism, amino acid metabolism, catalytic activity, multifunctional genes (MFO), glutathione-S transferase (GST), drug sensitivity, and cytochrome P450 related genes are upregulated in mutant type (M0, M-ipro) as compared to its wild type (W0, W-ipro), may be related to induce resistant in mutants of *B. cinerea* against iprodione.

## 1. Introduction

Grey mold disease caused by *Botrytis cinerea* is one of the most destructive disease on more than 200 plant species including various economically important crops like tomato, grapevines, pepper, cucumber and strawberry [1,2]. The annual economic losses of *B. cinerea* is more than $10 billion worldwide including fresh fruits and vegetables [3]. *B. cinerea* is a necrotrophic fungal plant pathogen of pre and post-harvest diseases with broad host range reproduces sexually and asexually [4]. *B. cinerea* produced micro and macro conidia on the surface of host plant cells [5,6]. These one-celled spores borne on multiple branches expressed its obvious symptoms as greyish to light brown mold leaves, stem, flowers and fruits of host plant [7]. Grey mold is more protruding during persistent rainy, heavy dew, and foggy weather around temperature 18–24 °C [8].

*B. cinerea* has a unique ability to survive in different environmental conditions in the form of conidia and seclerotia that make this fungus very destructive and hazardous of resistance development [1,9,10]. The tremendous results against *B. cinerea* have been attained by applying various agrochemicals on tomato, cucumber, vine grapes etc. Every year more than half billion-dollar cost of fungicides are sprayed against different pests [2]. Chemical control of grey mold currently approached by seven classes of fungicides including anilinopyrimidines (APs), dicarboximide (Dc’s), methyl benzimidazole, carbamates (MBCs), hydroxyanilides (HAs), quinone outside inhibitors (QoIs), succinate dehydrogenase inhibitors (SDHIs), and phenylpyrroles (PPs) [11,12]. Extensive use of agrochemicals induces resistance in plant pathogens in field. Nowadays we selected those site specific fungicides that have high efficacy, low toxicity and little human health risk [13]. However, these characteristics somewhat offset by their susceptibility to resistance development [14]. Iprodione, a dicarboximide (Dc) fungicide, has been used commercially for more than 30 years to control a wide variety of fungal pathogens. Among dicarboximides (Dcs), iprodione is a broad spectrum contact fungicide used as a foliar application and seed protectant for many fruits and vegetable crops. It has both preventive and curative action [15]. It was first manufactured in 1990. The mode of action of iprodione is to obstruct the synthesis of RNA and DNA during the germination of many fungal spores as well as lower the activity of enzyme NADH cytochrome c reductase, so the production of lipids and membrane restricted, ultimately mycelial growth inhibited. It can be used as a wettable powder, granules dispersed in water, flow able for all crops at the rate of 450–750 g/L [16].

Dc-resistant strains (iprodione) have been reported in many plant pathogenic fungal species [17]. Previously Hamada et al. [18] obtained iprodione resistant mutants of *Rhizoctonia cerealis* collected from wheat in China, *Botryosphaeria dothidea* from pistachio in California [19], *Alternaria* isolates collected from pistachio in California [20], and *B. cinerea* from strawberry and tomato in Hubei province, China [21].

The point mutation in the two components of histidine kinase genes (Bcos1) responsible for resistance to iprodione has been identified in *B. cinerea* isolated from strawberry fruit [21]. Substitution at codon I365 was dominant to cause resistance against iprodione among various strains of *B. cinerea* [22,23,24]. BcNoxA and BcNoxB catalytic subunits are responsible for pathogenicity and the formation of spores, which provide a favorable environment to fungal sclerotic to survive under adverse environmental conditions [25]. Various isolates of *B. cinerea* may possess low, moderate, or high levels of resistance to iprodione [26]. Mostly field isolates possess moderate to low level resistance, whereas laboratory mutants have high level resistance [27].

*B. cinerea* is a famous plant pathogen for its aptitude to become resistant against different fungicides. Although resistance against a different group of fungicides induced by different mechanisms like structural alteration in binding sites of pathogens reduces the affinity of fungicides, up regulation of fungicide target genes, decomposition of active ingredients reduces the efflux of fungicide concentration [28]. The study of genomics, transcriptome, bioinformatics and proteomic provide a new array to explore resistance attributes. Rendering RNA sequencing the most effective and direct way to explore resistance genes in mutant species of *B. cinerea*.

Guangxi province of China is one of the major tomato producing area [29] and facing several challenges of fungal diseases including *B. cinerea*. Iprodione, fludioxonil, and tebuconazole fungicides have been widely used against fungal diseases in Guangxi province for many years. Thus it is necessary to study the sensitivity of *B. cinerea* against these fungicides and asses the resistance in isolates for these chemicals before they are widely used to control *B. cinerea.* In this study, *B. cinerea* isolates collected from tomato plants in Guangxi Province, China. The key objectives of the present study were to: (i) determine the prevalence and frequency of *B.cinerea*. (ii) determine the iprodione sensitivity of *B. cinerea* isolates; (iii) preliminarily evaluate the risk of *B. cinerea* resistance to iprodione and to characterize the iprodione induced mutants; (iv) asses the fitness stability and pathogenicity of iprodione resistant mutants; and (v) investigate the molecular mechanism responsible for the development of resistance in *B. cinerea* against iprodione.

## 2. Material and Methods

### 2.1. Collection of Samples and Chemicals

There were one hundred and twelve different isolates of *B. cinerea* collected from different locations of Tian Dong, Tian Yang County, Baise City, Guangxi province, a southern region of China during 2016–2018 (Supplementary Table S1). Single spore isolation was accomplished from diseased leaves and fruits, as described by Fernández et al. [30]. All isolates were stored at 20 °C on dried filter paper [31]. Iprodione (96.7%) active ingredient (a.i) fludioxonil (99.2% a.i.) tebuconazole (97% a.i.); original drug, (Shandong Weifang Runfeng Chemical Co., Ltd., Jinan, Shandong, China) used in this experiment. Iprodione (0.103 g) was dissolved in acetone (10 mL) for the preparation of the stock solution and stored at 4 °C at dark for further use.

### 2.2. Sensitivity of B. cinerea to Iprodione

To evaluate the drug sensitivity, all strains preliminarily tested at 0.1, 1, 10, 100 µg/mL different concentrations of iprodione based upon previous findings of Grabke et al. [17]. The particular above mentioned concentrations in acetone were amended into PDA medium to examine the inhibitory rate. The inhibitory effect of acetone on mycelial growth of *B. cinerea* was 0.00001% in the sensitivity analysis of iprodione content which is ignorable. The mycelial plug of 5 mm diameter of 3d old *B. cinerea* colony was placed in the center of the 90 mm petri plate that contains iprodione amended media. These plates incubated at 23 °C for 3 days and radial mycelial growth (colony diameter) of each isolate measured in Petri plate by using a scale in the perpendicular direction and 5 mm original plug subtracted from the whole measurement. The experiment preliminarily repeated thrice, and each treatment had 3 replicates with control (only PDA medium). Those isolates that grew successfully on iprodione amended PDA were considered as resistant and failed as sensitive. Data processing system statistics (DPS version 7.05, Zhejiang, China) was used to analyze the effect of different concentrations of fungicides on *B. cinerea* mycelial growth inhibition rate to inhibit the rate of probability, the value of the ordinate (*y*), the concentration of the agent to the value of the abscissa (*x*), obtained virulence linear regression equation *y* = a + b*x*, effective medium concentration (EC_50_) value, and correlation coefficient (*r*).

### 2.3. Evaluation of B. cinerea Mutants Resistant to Iprodione

After measuring the colony diameter of *B. cinerea*, the plates were further analyzed to obtain resistant mutants induced by iprodione. The method of UV mutagenesis induced resistance into drug sensitive strains. The mycelial plug of two resistant strains was placed in 1 µg/mL PDA medium containing iprodione and incubated at 23 °C. After 3 days incubation, these plates put in preheated, 20W UV lamp, 25 cm irradiation. After applying the treatment, the plates were kept at the incubator in the dark for ten days. After observation, each isolate was transferred to a higher concentration of drug-containing culture plates and repeat the above UV-induced colonies taken at edge cake. The resistant strains were plugged into the PDA medium and continuously cultured for 15 generations and measured as described above. Only stable resistant mutants selected for further analysis (Supplementary Table S2).

### 2.4. Characteristics of Iprodione Mutants and Sensitive Isolates

Mycelial growth, sporulation, virulence, cell membrane permeability, osmotic potential, and enzymatic activities were performed to compare the fitness characteristics between the sensitive and resistant strains. Mycelial colony diameter and sporulation were assessed with or without iprodione amended PDA medium. The mycelial colony diameter measured perpendicularly after 24, 48, 96, and 72 h and sporulation after 12, 13, 14, and 15 days of incubation at 23 °C.

Mycelium growth assay was conducted on fungicide-free PDA. Mycelial plugs were cut from the borders of 3-day-old colony and transferred to the center of PDA plates. Four plates for each isolate were incubated at 23 °C in the dark and colony diameter was measured at two perpendicular directions after 60 h of incubation.

The virulence was assessed on detached tomato leaves as previously described by Fan et al. [32]. Fresh tomato leaves were washed with double distilled water then disinfected by dipping in 75% ethanol for 1–2 min followed by three washings of double distilled water and allow them to dry on filter paper at room temperature. Leaves were placed in a 15 cm petri dish and cover the petioles with a wet cotton ball for moisture. Each leaf was punctured with a sterile lancet (Yangzhou Shuangling Medical Appliance Co., Ltd., Shuangling, China) in the middle as previously applied by Fan et al. [32] than placed in 5 mm mycelial plug on top of the wounds. Lesion diameter recorded with the help of measuring tape from each leaf after 4 days of incubation in the dark at 23 °C.

Osmotic sensitivity was measured to evaluate the cell wall elasticity by adding 10, 20, 40, and 80 mg/mL NaCL in PDA medium after 3 days of incubation at 23 °C. Mycelial growth inhibition rate (MGIR) calculated by the formula MGIR (%) = (CK-N)/(CK-5) × 100, whereas CK (mm) is the control plate colony diameter, N (mm) is that of a plate containing NaCl amendment. To determine cell membrane permeability, the wild type and its mutant strains were first incubated in 100 mL potato dextrose broth in a conical flask. These conical flasks were placed in a continuous shaker at a temperature 23 °C for 3 days. A (5 mm) eight mycelial plug of *B. cinerea* was added in each 250 mL conical flask contains 20 mL solution of iprodione with a concentration of 0, 1, 5, and 10 μg/mL. The conductivities were detected after 0 h, 0.5 h, 1 h, 2 h, and 3 h with the help of DDS-11A conductivity meter (Nanjing T-Bota Scietech Instruments & Equipment Co., Ltd., Nanjing, China) after each treatment. The final conductivities were measured via boiling the mycelium in water for 5 min. Each treatment has three replications. The relative permeability was calculated by using this formula: Relative permeability (%) = (Ct − C0)/C × 100 whereas C0: initial conductivity value; Ct: conductivity value at a certain moment; C: After boiling treatment [14].

### 2.5. Enzymatic Activities of Iprodione Resistant Mutant and Its Corresponding Wild Type

Polyglacturonase (PG), Polymethylglacturonase (PMG) and Cellulase (CE) Performed by DNS (3,5-dinitrosalicylic acid) Method Previously Described by Jiang et al. [33]. All three isolates were grown on potato dextrose broth media (PDB) at 120 r/min Shaker culture at 23 °C for 3 days. 0.1 g mycelium was grounded in liquid nitrogen in precooled pestle and mortar. Add 5 mL of sodium acetate buffer (pH 5.5) and centrifuge at 16,000 rpm for 20 min. The supernatant was collected and stored at 4 °C for further enzyme analysis. The substrate used to estimate the PG activity was 1% polygalacturonic acid in 50 mM sodium acetate buffer. The reaction mixture contains 0.5 mL sample volume, 0.5 mL substrate and 1 mL sodium acetate buffer in an eppendorf tube. The mixture was incubated in water bath at 37 °C for 1 h. After incubation add 1.6 mL DNS and boil for 5 min. The absorbance was measured at 540 nm by using a spectrophotometer (Multiskan GO, ThermoFisher Scientific, Boston, MA, USA). The boiled enzyme was used as a control. The standard curve was drawn by taking different concentrations of galacturonic acid. The activity of CE and PMG was measured by using the above mentioned procedure except substrate. For PMG and CE 1% pectin added in 50 mM sodium acetate buffer and 1% CMC dissolved in 50 mM citric acid-sodium citrate buffer respectively. Total proteins were determined by coomassie brilliant blue method [34]. Standard solutions of proteins were prepared by Bovine serum mg/mL also used as control without sample (Supplementary Tables S3 and S4). 

### 2.6. Transcriptome Analysis of Wild and Mutant Strain

To explore whole sequence analysis, highly iprodione resistant mutant M0 and its wild type W0 were selected according to their fitness stability. Three biological repeats of wild type and mutant were grown in 100 mL of PDB under 0 µg/mL iprodione treatment grouped as (W0, M0). Similarly, three biological repeats of wild type and mutant were grown in 100 mL of PDB amended with 1 µg/mL iprodione and cultured at 220 rpm for 48 hr at 28 °C in the dark grouped as (W-ipro, M-ipro). After 2 days mycelia of all treatments were collected, washed with double distilled water, frozen in liquid nitrogen and stored in freezer at −80 °C. Untreated wild type (W0), mutant (M0) samples used as a control.

Total RNA was extracted according to the RNA isolation kit (TRIzole reagent, Invitrogen, Carlsbad, California, CA, USA). The purification of RNA was estimated with the Nano Photometer spectrophotometer at 260/280 nm (IMPLEN, California, CA, USA) and integrity was evaluated by assay kit (Nano 6000, California, CA, USA) using Bioanalyzer 2100 system (Agilent Technologies, California, CA, USA). The library generation and RNA sequencing was carried out by staff at I-sanger cloud platform. The further library was prepared by using an NEB-Next Ultra RNA illumina platform [35]. The Illumina platform converts the sequenced image signal to a text signal via CASAVA base calling and stores it in fastq format as raw data. Quality assessment was performed on raw data of each sample including base quality, base error rate and base level distribution statistics to obtain high quality clean reads by using FASTQ for subsequent analysis. The clean reads were mapped into a transcript and compared to a reference genome using Tophat2 alignment software. Some transcripts without annotation to the reference genome were called new transcripts.

#### Differential Gene Expression

We identified differentially express genes of two phenotypical groups of strains (Mutant and wild type, with or without fungicide) by using DEseq2 [36]. To estimate the gene expression level fragment per Kilobase of exon model per million fragments mapped (FPKM) tool was used. The statistical difference among genes was analyzed using the recommended Benjamini-Hochberg correction method (*p*-value ≤ 0.05) for controlling the false discovery rate (FDR). Eventually, the fold change (log fold_2_) and FDR values used as a key indicator the expression amount of different genes among samples and represent a heat map. Functional annotation of genes was performed as described by Wang et al. [14] and Cai et al. [37]. GO enrichment of differential expression of genes was implemented by the GO seq R packages (1.10.1) based on Wallenius’ non-central hyper-geometric distribution. KEGG pathways enrichment statistical analysis was performed by KOBAS software.

### 2.7. Quantitative Real Time PCR Analysis

For the confirmation of differential gene expression levels attained from the RNA sequencing data analysis, the qRT-PCR investigation was carried out. Total RNA was extracted from mutant and wild type with and without treatment of EC_50_ concentration of iprodione 1 µg/mL according to the kit instructions (TaKaRa Biotechnol. Co., Ltd., Dalian, China). For the preparation of reverse transcription first Single stranded cDNA was synthesized according to labelled kit instructions (TaKaRa Biotechnol. Co., Ltd., Dalian, China). The expression of seven genes were studied. Moreover, one control gene UBQ used as a reference gene. The primers were designed by using oligo software v7.37 and the specificity was confirmed by blast against *B. cinerea.* B.010 genome. The sequence of all primers were listed in (Supplementary Table S5). The length of primers fragment were between 19–23 base pair with melting temperature 80 °C. 1 µg RNA of each sample was first treated with RNase Free dHH_2_O and 4 × gDNA wiper Mix (Nanjing Nuo Weizan Biotechnology Co., Ltd., Nanjing, China) for removal of contaminated DNA in the extract. For the preparation of reverse transcription reaction system, the reaction mixture consisted of template cDNA 2 µL, reverse primer 0.8 µL (5 µM), forward primer 0.8 µL (5 µM) and ChamQ SYBR Color qPCR Master Mix 16.5 µL (TaKaRa Biotechnol. Co., Ltd., Dalian, China) of total volume 20 µL. The qRT-PCR reaction was conducted in a thermal cycler (ABI 7500, Hangzhou Langji Scientific Instruments Co, Ltd., Hangzhou, China) with initial temperature 95 °C for 5 min, 40 cycles include melting at 95 °C for 5 s annealing for 30 s and finally extension at 72 °C for 40 s. Three biological repeats of each treatment were performed with triplicate of each gene reaction vs. reference gene. Then changing in fold expression of different genes of the mutant and wild type was evaluated by using algorithm 2^−ΔΔCT^ value. All qRT-PCR data were analyzed by using Light Cycler^®^ 480 software version 1.5.1 (Roche Diagnostics Corporation, Indianapolis, IN, USA).

### 2.8. DNA Extraction, Cloning and Sequence Analysis of the Tubulin Genes

For DNA extraction wild type W0 and its mutant M0, mycelium was cultured on potato dextrose broth (PDB) and incubated for 48 h at 28 °C under shaking condition (200 rpm). Mycelia was harvested and washed with sterilized water and ethylenediamine tetra acetic acid (EDTA). The DNA was extracted by cetyl trimethylammonium bromide (CTAB) method [6]. The specific primers β-TUB (F-5′-TGAAGGTATGGACGAGAT-3′) (R-5′-GCATCCTGGTATTGTTGA-3′) under accession number (XM_001560987.1) and α-TUB (F-5′GTTGGAGTTCTGTGTCTA-3′) (R-5′GTGGTCAAGATGGAGTTA-3′) under accession number (XM_001555875.1) were used to amplify the complete coding sequence (CDS) of two Tubulin genes *bctubA* and *bctubB*. Three biological replicates of each strain used for DNA extraction and the PCR reactions were conducted three times independently for each sample. The amplified PCR products were purified using a PCR Purification Kit (TIANGEN, Beijing, China), ligated into the pMD18-T Vector (TaKaRa Biotechnol. Co., Ltd., Dalian, China), and then sequenced by Sangon (Guangzhou, China). The exon sequences of the *bctubA* and *bctubB* genes were translated into amino acid sequences and aligned using DNAMAN8.0 software (Lynnon Biosoft, Quebec, Canada) to check the mutation point.

### 2.9. Statistical Data Analysis 

All values related sensitivity, osmotic potential, enzymatic activities are mean of three replicates was analyzed using statistical software (DPS version 7.05, Zhejiang, China). The LSD test was used to determine significant differences (α = 0.05). Pearson’s correlation coefficients were calculated to evaluate the correlation of gene expression obtained by RNA-seq and qRT-PCR using Origin 9.0 software (Origin Lab, Newyork, USA). In the SAM method, the delta value was set to obtain an average. A false discovery rate (FDR) of 5% and the fold change cut-off value was established as 1.5. In LIMMA analysis, genes with a fold change >1.5 and *p* < 0.05 were considered as differentially expressed. Only the genes identified as differentially expressed by both SAM and LIMMA were considered.

## 3. Results

### 3.1. Sensitivity of B. cinerea to Iprodione

**Figure 1:**
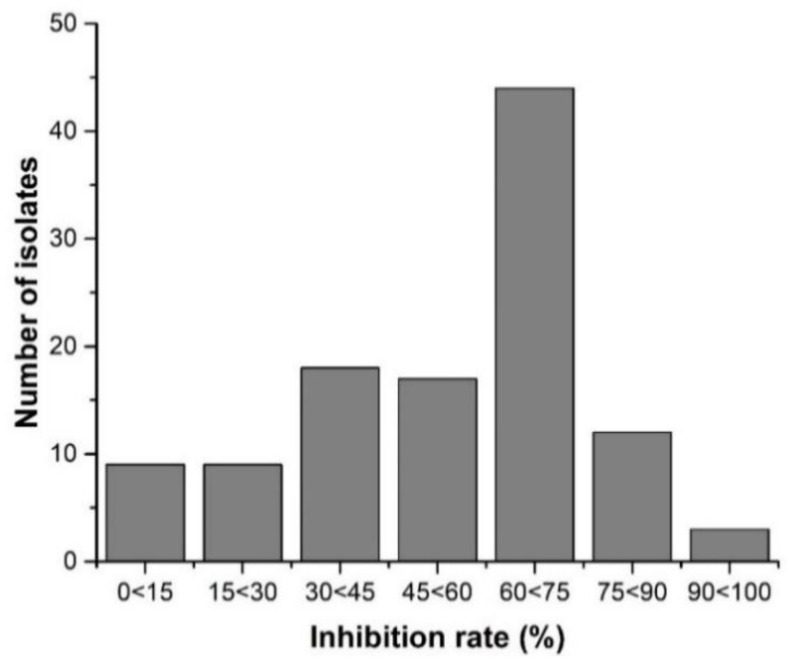
The inhibition rate of *B. cinerea* isolates against iprodione (0.01 µg/mL) collected from different areas of Guangxi Province China.

One hundred and twelve samples of *B. cinerea* were collected from different locations in tomato production area and tomato fields, Baise City, Guangxi Province, China. The sensitivity of B. *cinerea* was checked on PDA medium amended with iprodione at 0.01 µg/mL. The inhibition rate of all samples ranged from 7.69–74.35% with an average of 50.70% (Figure 1). The EC_50_ of iprodione against all samples ranged from 0.07 to 0.87 µg/mL with an average of 0.47 µg/mL, indicating these *B. cinerea* isolates were susceptible to iprodione. The value of EC_50_ of all isolates indicated that 0.47 µg/mL was an appropriate threshold concentration to assess iprodione resistance in the consequent experiments. Among them 5 isolates were highly sensitive to EC_50_ for 0.134 µg/mL iprodione and five isolates were moderately sensitive to EC_50_ for 0.434 µg/mL iprodione (Supplementary Tables S6–S9).

### 3.2. In Vitro Iprodione-Induced B. cinerea Mutants

UV radiation is a toxic mutagen and was expected to decrease the viability of the cells but also increase the probability of the emergence of mutants of drug sensitive strains. Five iprodione sensitive isolates of *B. cinerea* exposed to different concentrations of iprodione fungicide with 20W UV lamp were continuously cultured at 28 °C to induce rapid growth of mutants. The only strain B67 showed two mutants M1 and M0 respectively. The EC_50_ of these mutants were 674.48 μg/mL and 1025.74 μg/mL, respectively, and 597.63 and 906.94 times than that of wild isolate (EC_50_ was 1.12 μg/mL). However, other isolates showed higher sensitivity to iprodione and did not produce any mutant used as a control. These two mutants were continuously sub-cultured for 1, 5, 10, and 15 generations on drug free PDA medium for stability test (Supplementary Table S2).

### 3.3. Morphology and Physiology of Mutants

#### 3.3.1. Iprodione Resistant Mutant’s Mycelium Growth Rate and Sclerotia Formation

**Figure 2:**
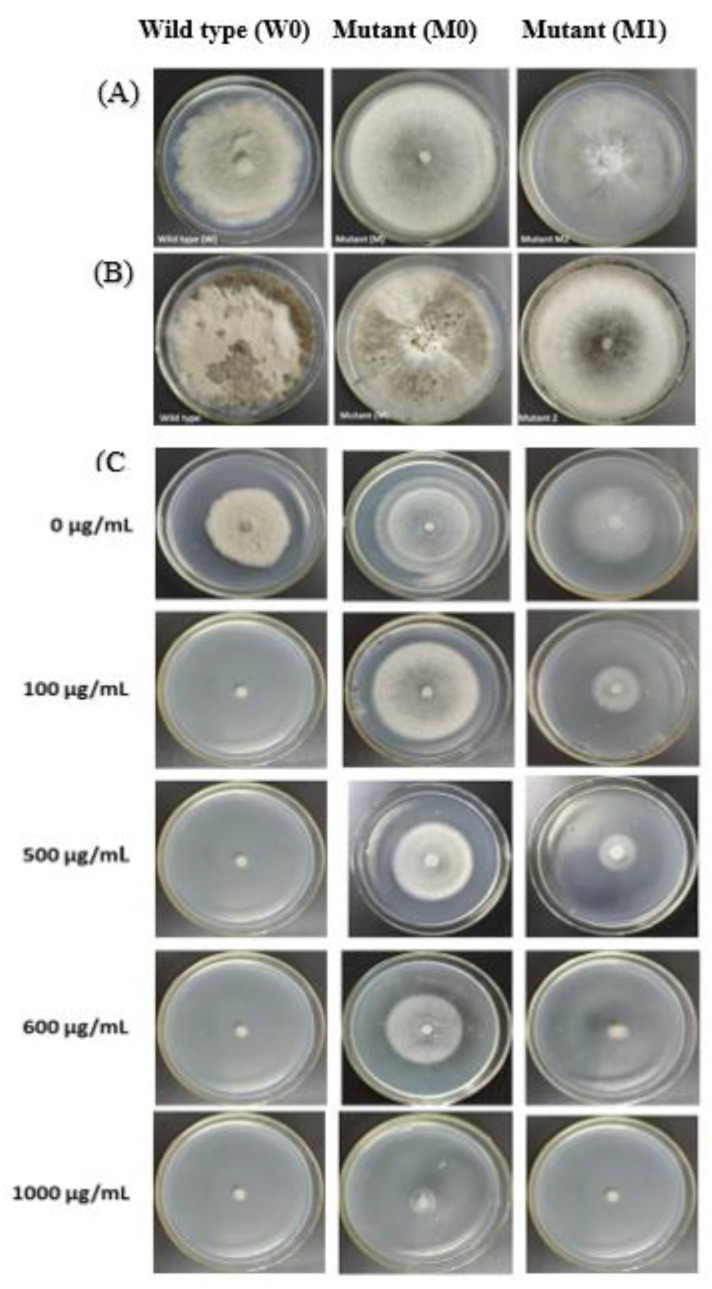
Colony morphology of *B. cinerea* Wild type (W0) and its mutant (M0, M2); (**A**) Mycelial growth of mutants (M0, M1) and its wild type on PDA medium after 5 days; (**B**) Sclerotia formation of wild type (W0) and its mutants (M0,M1) on PDA medium after 8, 12 and 14 days respectively; Sporulation: mean number (×10^6^) seclerotia per square centimeter, (**C**) Mycelial growth of wild type (W0) and its mutants (M0, M1) after exposed 100, 500, 600 and 1000 µg/mL to iprodione on PDA medium at 28 °C for 3 days.

The results showed that mycelial growth of wild type on PDA medium is significantly higher than both mutants after 5 days at 28 °C (Figure 2A). Wild type (W0) showed maximum sclerotia formation on PDA after 8 days; in petri dish edges are produced around a small contiguous black sclerotia; did not spread throughout the surface of the medium. M0 produced dark grey to black sclerotia after 12 days and spread over the medium and M1 after 14 days produced sclerotia over the surface of the medium (Figure 2B). The mutant M1 after 10 days began to produce small contiguous black sclerotia circles at the edge of the dish and after 12 days spread on the petri dish. When PDA medium was amended with 100, 500, 600, and 1000 µg/mL iprodione, mutant M0 showed significantly high mycelial growth than M1 and wild type W0 (Figure 2C). Mutant M0 after 12 days began to produce fewer black sclerotia circles at the edge of the dish with 5 µg/mL iprodione containing media. In contrast, wild type failed to produce any (Supplementary Figure S1).

#### 3.3.2. Cell Membrane Permeability Osmotic Sensitivity Pathogenicity and Enzymatic Activity of Mutants and its Wild Type

**Figure 3:**
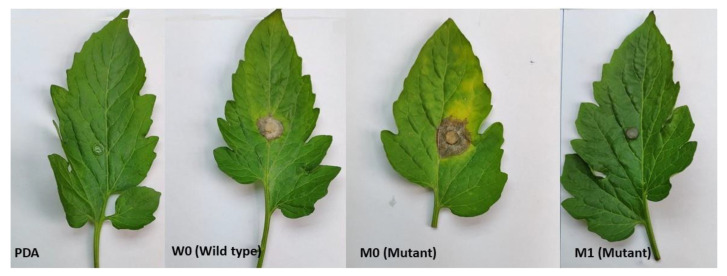
Virulence of *B. cinerea* Wild type (W0) and its mutnats (M0, M1) on detached tomato leaves after 24 h of inoculation.

Cell membrane permeability at four different concentrations (0, 1, 5, 10 µg/mL) of iprodione were measured, the relative rate of infiltration with the extension of the processing time increases and gradually stabilized in M1, indicating that the cell membrane permeability is significantly higher in M1 and wild strain than M0 (Supplementary Figure S2). Whereas, there was a no significant difference in osmotic potential of wild type and its mutants (Supplementary Figure S3). Protein concentration was calculated according to the sample suction photometric method. The remarkably highest protein contents were in W0 (1023.97 µg/mL) than M0 and M1 mutant (941.38, 908.02 µg/mL respectively (Supplementary Table S10). Enzymatic activities of both mutants and wild type were also measured. Although PG and CE activity was higher in M0 than M1 and wild type W0. (Supplementary Table S11). In pathogenicity assays, detached tomato leaves inoculated with both mutants (M0, M1) or wild type strain showed typical symptoms and lesions by W0 and M0, while the only PDA or control plants and M1 remained asymptomatic after 24 h of inoculation (Figure 3). These results showed that mutant M0 pathogenicity and enzymatic activity is more vulnerable than M1.

#### 3.3.3. Cross Resistance

The sensitivity of both *B. cinerea* mutants (M0, M1) were also determined against tebuconazole and fludioxinil using a discriminatory dose. Both iprodione mutants showed positive cross resistance against these fungicides.

### 3.4. Transcriptomic Data Analysis of Iprodione-Resistant Mutant and Its Wild Type after Exposed to Iprodione In Vitro

The results revealed that transcriptomic sequencing of twelve iprodine resistant mutant and wild type samples generating 187,062,138 raw reads after the screening and filtration of raw reads, a total of 186,026,964 clean reads were obtained. The percentage nucleotide quality score of more than 20 (Q20) was noted as high as 97.75%, and the percentage of Guanine and Cytosine GC (%) among all nucleotides was obtained as 47.33% (Table 1). According to Illuminia platform, the contrast efficiencies of mapped reads, uniquely mapped reads and multi mapped reads were 95.91%, 0.52%, and 95.2%, respectively, as compared to the reference genome (Table 1). A total number of transcripts was 20,375 and its length varied from 201 to1800 bp with an average 1000 bp (Supplementary Figure S4). On the basis of existing reference genome based assembly is performed by using compare software. To check the presence of novel transcripts, we combined the RNA-seq data of 4 samples with 3 biological repeats to identify novel transcripts, which are not assembled in the database. New transcript is obtained by comparing it with known transcript and further classified into 12 different class codes (Supplementary Figures S5 and S12). Out of 20,375 transcripts, 13,639 complete matches of intron chain, 3574 potentially novel isoforms, 1229 unknown intergenic transcript, and 372 generic exonic overlaps with a reference transcript were obtained during analysis. The raw data is submitted to NCBI under SRA number SRP254522.

#### 3.4.1. Functional Annotation of Transcripts and Unigenes

Almost all transcripts (13,703) and unigenes (11,698) sequences were alligned to the NCBI and annotated at least one of these six databases, Non Reductase (NR), SwissProt, Protein family (Pfam), Gene ontology (GO), Clustre of Orthologous Groups of Proteins (COG) and Kyoto Encyclopedia of Genes and Genomes (KEGG) (Supplementary Figure S6). The database indicated that maximum transcripts (13,687; 99%) and unigenes (11,685; 99%) were alligned by NR, whereas more than 50% of transcripts and unigenes were aligned to the COG, Pfam, and Swissprot databases. The minimum number of transcripts vs. unigenes was annotated by KEGG 33%. Moreover, 14 transcripts and 12 unigenes remained unannotated (Supplementary Table S13 Excel sheet).

#### 3.4.2. Discovery of New Genes

**Figure 4:**
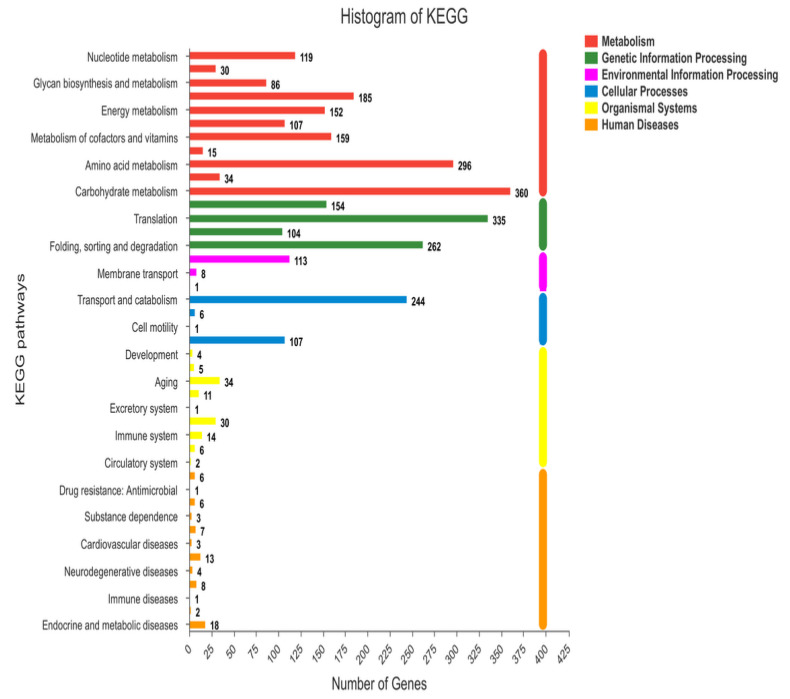
Histogram of KEGG pathways.

A total of 1024 new genes were discovered according to the above mentioned six databases. COG annotated 150 new genes in 14 different categories, GO 160 new genes in 19 different compartments (Figure 4), and KEGG 17 in 12 different disciplines. Furthermore, GO database is secondarily classified into three categories, namely molecular functions (47), cellular components (57), and biological processes (56) of new genes were explored. The highest number of new genes were involved in binding, metabolism, and cellular processes. COG annotated a maximum 84 new genes, which were poorly characterized, and 44 genes were involved in the repair, replication, and recombination of RNA. KEGG aligned the highest number of genes in the biosynthesis of the secondary metabolism process.

#### 3.4.3. Differential Genes Expression of Iprodione Resistant Mutant and its Wild Type After Exposure to Iprodione

After obtaining the clean reads, the differential expression of unigenes was analyzed by using software DESeq2. A total of 281 unigenes were expressed in mutant type (M0), including 166 up-regulated and 115 downregulated with or without iprodione treatment. Meanwhile, wild type (W0) showed 99 unigenes expressions in which 85 were up-regulated and 14 downregulated (Table 2). 

**Figure 5:**
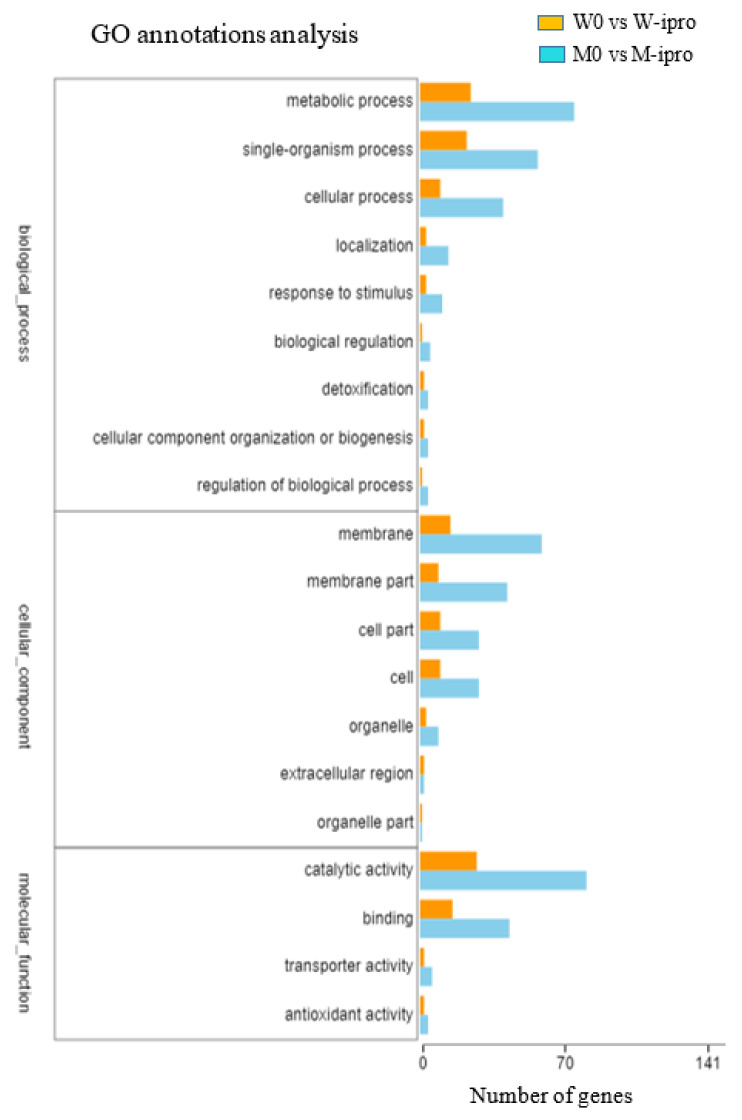
Histogram showed a significant difference (*p*-value < 0.001) in W0 vs. W-ipro and M0 vs. M-ipro by GO annotation. The X-axis represents the number of genes and Y-axis represents the three GO terms under biological processes, cellular component and molecular function.

The analysis showed that mutant and wild type shared 19 DEGs and 262 and 80 unique DEGs were detected in mutant and wild type respectively when exposed to iprodione. Overall, 1897 DEGs were detected after iprodione exposure and 1707 without iprodione between mutant and wild type. Furthermore, M-ipro vs. W-ipr0 and M0 vs. Wo share 1192 common unigenes and 720 unigenes are upregulated in mutant corresponding to wild type after iprodione treatment (Supplementary Figure S7). These results demonstrated that the DEGs pattern significantly changed in mutant and wild type with or without iprodione exposure, suggesting that some compounds may be specific to produce resistance in mutants against iprodione treatment.To understand the mechanism of resistance in *B cinerea* against iprodione, the gene function, expression level, and expression difference were analyzed in gene set analysis. Genes of the same function were located on one transcript, particularly within the three loci easy to annotate by gene ontology (GO) rather than those situated on different transcripts. The genes related to metabolic process, localization, ATP binding, transmembrane transport antibiotic activity, and the cellular process were most abundant in mutant type (M-ipro vs. M0) relative to wild type (W-ipro vs. W0) (Figure 5).

**Figure 6:**
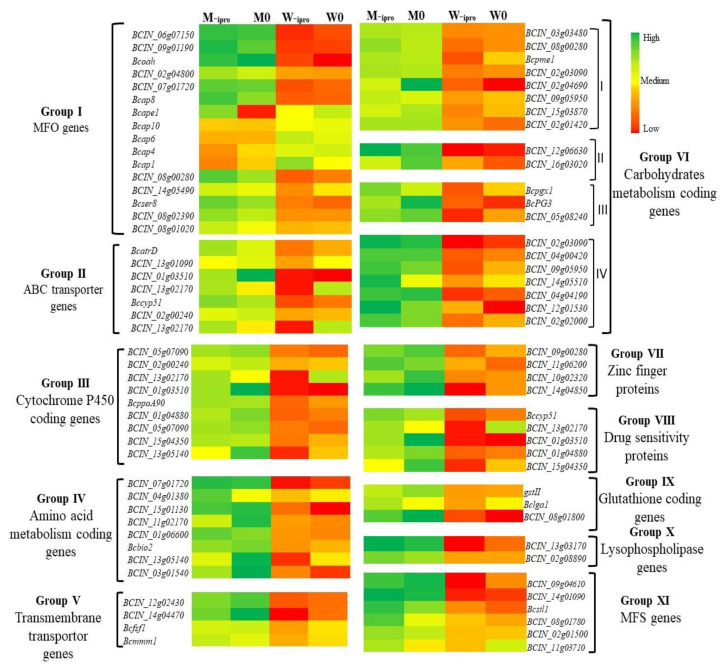
Variability in altered genes of wild type and its corresponding mutant with or without iprodione application represented in the form of Heat map. Genes with the same annotation modulated in a similar group. Twelve major groups are displayed in the heat map (Group I, II, III, IV, V, VI, VII, VIII, IX, X, XI, XII). Group VII have four subgroups I (Glycosyl family), II (Cellulase genes). III (Polygalacturose genes) and IV (carbohydrate metabolism).

To elucidate the difference between mutant and wild type, the expression pattern of four treatments were divided into a hierrachial clustering analysis (Supplementary Figure S8). Out of 1912 genes, we focused our attention on highly expressed genes in M-ipro that were more or less related to iprodione resistance and assembled them into 12 small clusters according to their functions (Figure 6). In clusture I, MFO (multifunctional genes) were analyzed (*BCIN_06g07150*, *BCIN_09g01190*, *BCIN_07g01720*, *BCIN_02g04800*, *Bcape1*) highly expressed in M-ipro and involved in molecular and biological functions of *B. cinerea*. Among the five genes of aspartic proteinase family (*bcap1*, *bcap4*, *bcap6*, *bcap8, bcap10*), only *bcap8* log2 value was significantly high in M-ipro, while the remaining genes did not show any significant difference in both mutant and wild type with or without iprodione treatment. In cluster II genes, set ABC transporter genes (*BcatrD*, *Bcbfr1*) were highly expressed in mutant type M-ipro than M0 and downregulated in wild type (W-ipro, W0). Various cytochrome p450 coding genes were expressed in a comprehensive data base and assembled in cluster III. Almost all genes were depressed in wild type (W-ipro, W0). *BccpoA90*, *Bccyp51*, and *BCIN_15g04350* expression was high in M0 and M-ipro (Figure 6) except *BCIN_02g00240*. Genes that were involved in amino acid metabolism exhibited high variability among mutant (M-ipro, M0) and wild type (W1, W0). Cluster IV has transmembrane transporter genes which were also expressed in both wild and mutant type. Only *BCIN_14g04470* and *BCIN_12g02430* were highly downregulated in W-ipro and W0 and upregulated in M0 and M-ipro.

In contrast, many more genes had no significant difference among all treatments (Supplementary Table S14 Excel sheet). Moreover, the maximum high expression was detected in carbohydrate metabolism, glycosyl family, polyglacturose family and cellulose related genes attributed in cluster VI. *BCIN_02g04690* log fold 2 values significantly high in M0 and low in W0 related to carbohydrate binding. In cluster VII several genes were upregulated belongs to zinc finger proteins in mutant type (M-ipro, M0). Intriguingly *BCIN_10g0230*, *BCIN_09g00280*, and *BCIN_14g04850* genes were highly upregulated in M0 rather than M-ipro and downregulated in wild type (W-ipro, W0). Drug sensitive proteins were highly downregulated in wild type after exposure to iprodione pooled in cluster VIII. *BCIN_15g04850* and *BCIN_13g05140*, highly upregulated in mutant without iprodione treatment instead of its application (Figure 6). Glutathione-S transferase encoding genes, i.e., GSt enzymes play an important role to detoxify the chemicals. *BCIN_08g01800* gene regulating glutathione enzyme was more expressed in mutant without iprodione application than wild type. Clusture X, XI represents the lysophospholipase and super family genes (MFS). Two sugar transporter genes (*BCIN_09g04610*, *BCIN_14g01090*) of super family upregulated after exposure to iprodione fungicide in the resistant mutant. *BCIN_13g03170* lysophospholipase gene expression log fold 2 value was −0.629 in W0, showed decrease −1.072 in W-ipro while expression level was increased in mutant after exposure to iprodione (Table 3). On the basis of these results, the presence of resistance in mutant strain may not be due to particular resistant gene against chemical or inactivation of enzymes and metabolic process. These data base also suggested that synergistic and combination of several genes belong to different functions or families generate resistance in mutants against a particular drug or multi drugs.

### 3.5. qRT-PCR Amplification of Some Specific Genes

**Figure 7:**
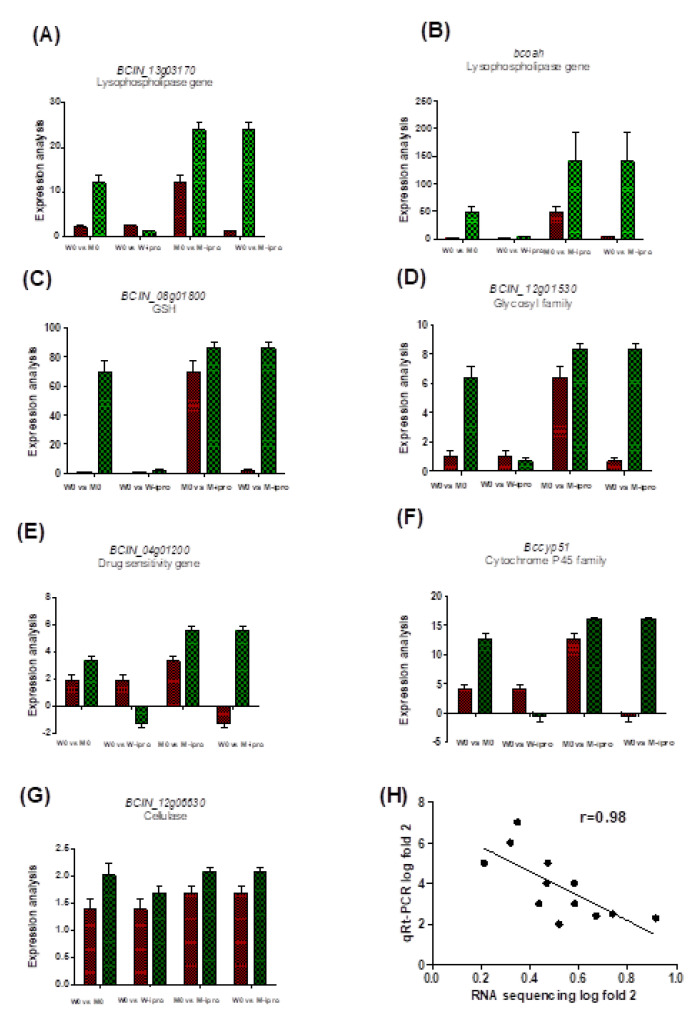
Validation of seven DEG’s log fold 2 value of RNA seq by qRT-PCR of wild type and its corresponding mutant with or without iprodione treatment. (**A,B**) lysopholipase genes, (**C**) Glutathione-S transferase gene, (**D**) Glycosyl family carbohydrate metabolism, (**E**) Drug sensitivity genes, (**F**) Cytochrome P450 family, (**G**) Cellulase genes, (**H**) Pearson correlation of log fold 2 value of qRT-PCR and RNA sequencing of wild type and its mutant after iprodione application. The mRNA abundance was normalized by using the reference gene UBQ and relative expression (log fold 2) was valued as 2^−ΔΔCT^. All values of qRT-PCR represents as mean ± SD (*n* = 7).

In order to verify the transcriptomic analysis of mutant and wild type *B. cinerea*, a total of eight highly expressed encoding genes of lysophospholipase (*BCIN_13g03170*), drug sensitivity (*BCIN_04g01200*), cytochrome p450 (*Bccyp51*), cellulase (*BCIN_12g06630*), glutathione-S- transferase (*BCIN_08g01800*), oxaloacetate acetyl hydrolase (*bcoah*), cellulase (*BCIN_12g06630*), glycosyl family (*BCIN_12g01530*), and reference gene *UBQ* have been selected for expression analysis of RT-qPCR. Among these genes gluthathione-S transferase gene (*BCIN_18g01800*) highly upregulated in mutant type with or without iprodione application (Figure 7C). Moreover, two lysophopholipase genes (*BCIN_13g01370*, *bcoah*) expression in the mutant (M-ipro) were highly upregulated as compared to M0 and wild type (W-ipro, W0) (Figure 7A,B). The drug sensitivity and cytochrome familyP450 genes (*Bccyp51*, *BCIN_14g01200*) were downregulated in wild type after iprodione treatment (Figure 7E,F). The cellulase gene (*BCIN_08g01800*) was highly expressed in all treatments (Figure 7G).

### 3.6. Detection of Mutations in Tubulin Genes in Iprodione Mutants

**Figure 8:**
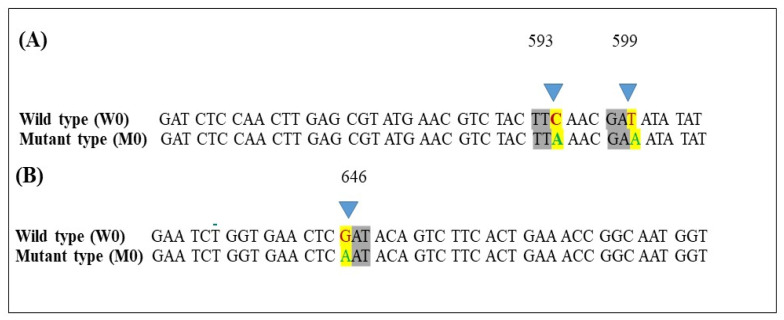
Amino acid sequence alignment of *B. cinerea* tubulin genes in wild type (W0) and its mutant (M0), (**A**): Mutation variation in *bctubA* gene at position 593 and 599, (**B**): Mutation variation in *bctubB* gene at position 646.

Genome sequencing of wild type (W0) and its mutant (M0) showed two tubulin genes encoding *bctubA* and *bctubB*. The coding region of *bctubA* had 1985 nucleotides encoding 661 amino acids, which was 65% match with *bctubA* B05.10 strain of *B. cinerea* (Gene Bank accession number: XP_024546500.1). While the coding region of *bctubB* gene had 1919 nucleotides encoding 639 aminoacids was match with *bctubB* (*B. cinerea* B05.10) (GeneBank accession number: XP_024546928.1). The *bctubA* gene in mutant (M0) replaced TTC and GAT codon at position 593 and 599 by TTA and GAA, resulting in replacement of phenyl alanine into leucine (transversion C/A) and aspartic acid into glutamic acid (transversion T/C) respectively. Whereas, in *bctubB* gene GAT codon at position 646 replaced by AAT and aspartic acid converted into asparagine (transition G/A).(Figure 8) No point mutation was found in wild type as compared to control (*B. cinerea* B05.10 strain).

## 4. Discussion

Fungicide resistance in different pathogens has been a major problem in crop protection worldwide in two decades. The extensive use of fungicides to control pathogens in same area for several seasons creating this problem; as a result the efficacy of fungicides decreasing and resistance induced into the pathogens against a specific or multiple fungicides is increasing. Therefore, it is necessary to evaluate the resistance risk in the lab before new fungicides are widely practice into the field. In Guangxi province, grey mould, caused by the fungal pathogen *B. cinerea*, is one of the most devastating tomato diseases, and the control of this disease is mainly by the application of chemicals. In the present study, 112 isolates of *B. cinerea* collected from major tomato production area of Guangxi province, China and screened with different concentrations of iprodione and found that toxicity steadily increased in mutants. This is considered as the first report to assess iprodione sensitivity of *B. cinerea* collected from Guangxi Province China. Previously sensitive isolates of *B. cinerea* on tomato were detected in Germany [38]. All strains were sensitive to iprodione from 0.07 to 0.87 µg/mL with an average of 0.47 µg/mL. These results are similar to *B. cinerea* collected from strawberry, a procymidone and zoxamide sensitive strain from Hubei province having EC_50_ value of 0.25 µg/mL and 0.360 µg/mL, respectively [22]. No 100% sensitive strain was discovered in our collected samples because iprodione is a site specific fungicide with high efficacy, less toxicity and lower application rate. The high dozes or sustained application of fungicides leads to put selection pressure to develop fungicide resistance [39]. In order to solve this problem, there was an urgent need to develop effective resistance management strategies.

High frequencies of *B. cinerea* resistant isolates have been recorded not only iprodione but also to various groups of fungicides including DC, MBC, and PP across the globe [40,41,42]. Although field resistant mutants of iprodione was documented in Northeatern part (21%), Henan (8%), and also in Anhuai (5%) of China [18,43]. This study was showed low frequency of resistant mutants as compared to the previous one, but with the passage of time resistance level will increase with rapid and continuous application of DC’s fungicides. In the present work, UV irradiation used to detect specific iprodione resistant mutants to assess the risk from emergence strain. After stability and sensitivity testing the EC_50_ of UV mutants (M0, M1) exceeded 1 µg/mL which was higher 1025.74 μg/mL and 674.48 μg/mL respectively than corresponding wild strain (W0). The cell membrane permeability was significantly increased in M1 and wild type than M0 which indicate M0 is more stable. In contrast CE and PG enzyme activity were higher in M0. Polygalactronase family genes (*Bcpgx1* and *Bcpg3*) involved in pathogenicity expression were higher in M-ipro than wild type. These findings were distinction with the Guo et al. [44] found that resistant strains when continuously dealt with fungicides, pathogens lose its potential and viability resulted in failure of infection. The virulence of M0 like as wild type while M1 results supported the previous findings of Chen et al. [45] mutant strain of *Verticillium dahliae* lose its pathogenicity by the repressed of cytochrome p450 gene expression. Comparative genomic studies have been frequently conducted to understand the expression of selected genes against different chemicals. However, inadequate information is available due to a lack of whole genomic sequencing (RNA seq data) and suitable methodologies for comparative transcriptome. In recent studies, transcriptome analysis has been widely applied on fungicidal resistant plant pathogens, including Fusarium spp. [40], *B. cinerea* [37], and *Penecillium didgitatum* [46]. According to our transcriptome analysis the expression of lysophospholipase genes, transmembrane transporter genes, MF (multifunctional genes), MFS (super family genes) encoding, amino acid and carbohydrate metabolism genes were clearly upregulated in field mutant (M0_,_ M-ipro) than wild type (W0, W-ipro) with or without application of iprodione (Supplementary Table S15 Excel sheet). Many microorganisms produce phospholipases heterogeneous groups of enzymes, either secreted or induced intracellularly by physical disruption of the cellular membrane [47]. Among them lysophospholipases are key enzymes that hydrolyze the esters linkages in glycephosopholipids and contribute to detoxification of potentially cellular lysophospholipids that facilitate the survival of fungi in vivo, cell wall integrity, proliferation expression of virulence, fungal cell signaling and immunomodulatory pathways [48]. Here, the expression of lysophopholipase genes (*BCIN_13g03170*, *BCIN_02g08890*) were superiorly upregulated in M-ipro vs. W-ipro than M0 vs. W0 (Figure 7) contribute to iprodione resistance. *BCIN_02g08890* is highly upregulated in mutant strain with or without fungicide and absent in wild type.

Plant cell wall mainly composed of polysaccharides with less amount of glycoproteins esters, mineral contents, phenolic compounds and enzymes [49]. The predominant polysaccharides are cellulose, hemicellulose galacto (mannans, xylans, and xyloglucans), and pectin. Carbohydrate-degrading enzymes of pathogens constitute a key factor involved in the metabolic breakdown of glycoconjugates, oligosaccharides, and polysaccharides of host plant cell wall components during infection or invasion [50]. Cell wall degrading enzymes are abundantly found in *B. cinerea* [51]. Recent studies revealed that cellulase, xylanase, and pectinase (glucanase) enzymes functioned as a virulence factor in phytopathogens and were recognized as PAMPS by plants to trigger the PTI responses, during host plant–pathogen interactions [52].

In this study, we analyzed the enzymatic activity of cellulase, polygalactrose, and polymethylgalactrose in mutant and wild type *B.cinerea*. Enzymatic assays and gene expressions of cellulase (*BCIN_12g06630*, *BCIN_16g03020*) and xylanase A (*BCIN_03g03480*, *Bcxyn11A*) were more upregulated in mutant than wild type (Figure 7). Cellulase catalyzes the degradation of the β-1,4- glycosidic bonds in cellulose [53]. Cellulase is an elicitor in plant–pathogen interactions but its enzymatic activity is independent of its elicitor. In contrast, it was previously reported that xylanase activity promotes the necrotic infection of *B. cinerea* into plant tissues [54]. *Bcxyn11A*, an endo-β-1,4-xylanase degrades plant cell wall xylan contents, and is required for successful infection. Furthermore, a small nanogram of xyn A was sufficient as an elicitor in *S.lycopersci* and *N. benthamiana* [55]. In contrast, not all fungal xylanases have been conclusively involved in pathogenicity and virulence [51]. The *B. cinerea* equipped with different patterns of endopolygalactrase (*Bcpg*) genes and exopolygalactrase (*Bcpgx*) genes to degrade pectate machinery of host cell. In the current study, the expression of *Bcpg2* and *Bcpgx1* genes were highly upregulated in (M-ipro vs. W-ipro) and there is no change in *Bcpg1* and *Bcpg4* in all treatments. *Bcpg2* is a necessary gene during primary infection and lesion expansion in tomato [56]. A major result of our work is reported that there is no diversification in virulence genes of mutant and wild type before and after iprodione application. *B. cinerea* secretes several genes of the aspartic proteinase (AP) family to perform proteolytic activity. A functional analysis of our results showed that there is no change in the expression of *Bcap* genes of wild and mutant type after iprodione application (Figure 6). No significant difference was found in *Bcap1-5* genes mutants and the wild type strain of B05.10.

The resistance of fungi is sturdily associated with multiple mechanisms, including (1) the nonsynonyms mutation in the target protein encoding genes, (2) the upregulation of the target proteins, and (3) the overexpression of transporting and membrane encoding genes. Fungal efflux pumps such as cytochrome P450 are the most versatile natural bio-catalyst genes and constitute a large superfamily related to the detoxification of fungicides, insecticides, and xenobiotics under mild conditions [57,58].

The mechanism of resistance to iprodione was associated with point mutations in the tubulin gene that changes the structure of the fungicide binding site to decrease sensitivity according to Grabake et al. [28]. According to the genome sequencing information, two tubulin genes showed mutation. A point mutation at codons 593 (A198E) and 599 (F200Y) in *bctubA* gene and at 646 codon (R216C) in *bctubB* were detected from resistant strain M0, and a similar mutation was reported in a field isolate of *B. cinerea* resistant to benzimidazole that had a mutation at points A198E and F200Y [59]. A novel point mutation at codon 646 (R216C) was detected in *bctubB* in mutant (M0). Contrarily, point mutation variations at single codon I365S/N/R of the *Bos1* gene were responsible for dicarboximide (iprodione) low resistance, as reported from France, England, Israel, Japan, New Zealand, Italy, Switzerland, and the United States. Iprodione reduced DNA, RNA synthesis in the germinating fungal spore and inhibited the enzymatic acivity of NADH cytochrome c reductase, thereby preventing lipid and membrane synthesis and ultimately mycelium growth [60]. Wang et al. [14] reported that the resistance in *B. cinerea* to fenhexamid mainly relied on the mutation of *BCIN_16062* encoding P450 gene. In a hypersensitive strain of *Candidas albicans*, *CaALK8* gene was promoted and confers multidrug resistance [61]. The expression of *BCIN_01g03510* and *BCIN_13g05140* were upregulated in mutant (M0) relative to wild type (W0, W-ipro) (Figure 6). Glutathione S- transferases are multifunctional detoxification enzymes that regulate the cell functions, countering oxidative stress and signal transduction with several resistance mechanisms [62,63]. Our findings revealed that *BCIN _08g01800* was highly downregulated in wild type (W1-ipro) after iprodione application while other genes showed similar expression. Remarkably, transmembrane proteins in fungal efflux systems (ABC and MFS transporter genes) have been reported to provide protection for fungal cells against antibiotics and fungicides found in the environment [64]. Furthermore, these transporters determine the baseline of sensitivity or resistance to fungicides [65]. Several genes encoding transmembrane transporters were identified from the RNA sequencing data (Figure 6). Most of them showed higher expression in mutant strain than wild type. Particularly, two MFS encoding genes *Bcstl1* and *BCIN_08g01780* were highly upregulated in mutant (M-ipro) after iprodione treatment than M0 and wild type (W-ipro, W0). Intriguingly, *BCIN_14g01090* gene logfold2 value were intensely upregulated before and after iprodione application in mutant (M-ipro, M0) and downregulated in (W-ipro, W0). The high expression of drug efflux transporter has been reported in different isolates of *B. cinerea* against different classes of fungicides [66,67]. On the other hand, Grabke et al. [17] reported the overexpression of MFS encoding genes of *B. cinerea* conferring a low level of resistance to iprodione in strawberry. Contrarily, MFS genes contributed to resistance in *Fusarium* spp against prochloraz and carbendazim fungicides [40,46]. Likewise, *BCIN_14g04470* and *BCIN_12g02430* in fungal efflux were highly upregulated in mutant strain (M0) relative to wild type (W0).

## 5. Conclusions

The risk of resistance selection in *B. cinerea* due to the extensive application of DCs (iprodione) may be a severe problem in Guangxi Province China. In the current study, the results showed that *B. cinerea* isolates were sensitive to iprodione The laboratory UV induced resistant mutants were obtained through continuous iprodione treatment, which indicated that *B. cinerea* quickly adopt resistance to iprodione. The resistant mutant demonstrated an EC_50_ value 1025.74 μg/mL higher than curative dosage. The resistant mutant had a high level of fitness (mycelial growth, sclerotia formation and aggressiveness) as compared to sensitive isolates. We identified two mutation points at codon 593 (A198E) and 599 (F200Y) in the *bctubA* gene, including one novel point mutation in *bctubB* gene at position 646 codon. Our research work also provided a comprehensive molecular mechanism involved in the *B. cinerea* resistant mutant. By analyzing RNA sequencing data for wild type and mutant with or without iprodione application, genes related to iprodione resistance were highlighted and identified. These DEGs were involved in the production of detoxification enzymes, metabolism, catalytic activity, MAPK signaling pathway, drug efflux, and transporter functions to resist the chemicals. Resistant management strategies should be implemented to delay the spread of iprodione resistant mutants in the field. Furthermore, integrated disease management strategies should be practiced followed, including the use of biological control and agricultural practices.

## Figures and Tables

**Table 1:** Details of raw and clean data of twelve transcriptomes of *B. cinerea* and the reference genomes.

Strain	Raw Reads	Clean Reads	Clean Bases	Mapped Reads	Q 20 Avg (%)	GC Avg (%)
M0	47,684,718	47,218,908	7,057,947,521	44,704,018 (95.8%)		
M-ipro	45,648,745	46,083,059	6,885,980,894	44,251,477 (96%)		
W0	47,109,858	46,628,979	6,970,197,563	44,597,601 (95.62%)	97.76	47.23
W-ipro	46,618,817	46,096,018	6,885,797,287	44,052,162 (96.22%)		
Total	187,062,138	186,026,964				

**Table 2:** The total number of DEG’S in wild type and its mutant with or without iprodione.

Strain	All DEG	DEG Upregulated	DEG Downregulated
M-ipro vs. W-ipro	1897	886	1011
M0 vs. W0	1707	890	817
M-ipro vs. M0	281	166	115
W-ipro vs. W0	99	85	14

**Table 3:** Major genes related to resistance in *B. cinerea* wild type and its mutant with or without iprodione application.

Gene ID	Log_2_ Fold Change Value				Function Annotation
	M-ipro vs. W-ipro	M0 vs. W0	M-ipro vs. M0	W-ipro vs. W0	
*BCIN_02g08890*	9.104	8.746	0.565	0	Integral cellular component domain
*BCIN_13g03170*	8.222	5.529	0.378	−0.231	Glycerophospholipid metabolism
*BCIN_06g07150*	6.213	5.503	0.12	−0.599	Energy production and conservation
*BCIN_09g01190*	4.878470612	6.006	−0.511	1.2044	Zinc finger proteins
*BCIN_13g04220*	8.618	−7.08119	8.576	−21.308	Chaperones proteins
*BCIN_08g06520*	7.317	4.154955	1.07079	−4.216	Oxygenase super family
*Bcpg2*	4.978	−0.967	2.896255	−2.978	Glycosyl hydrolase family
*Bcoah*	4.878	6.600	−0.511	1.204	Isocitrtae lyase family
*BcatrD*	2.529	1.66	0.266	−0.249	ABC transporter proteins
*Bclga1*	2.266	0.745	0.865	−0.705	Glutathione S transferase proteins
*BCIN_12g0243*	2.597	3.064	−0.407	−0.046	Catalyze aminotransferase acid
*Bcrds2*	1.229	1.092	−0.062	−0.192	Drug sensitivity proteins
*Bcltf1*	0.531	1.292	−1.108	−0.3241	Sexual development transcription factor NsdD
*BCIN_06g02680*	2.00	0.835	1.292	−1.108	Phenylalanine aminomutase enzyme
*BCIN_08g02390*	2.837039	2.628415	0.436	0.223	Heat shock binding proteins
*BCIN_02g01500*	1.929	0.199	0.318	1.779	Carbohydrate transport and metabolism
*BCIN_04g01200*	3.721	2.744	1.035	0.061	Steroid biosynthesis proteins
*BCIN_08g01540*	2.119	1.637	0.0715	−0.411	Steroid biosynthesis proteins
*BCIN_03g03480*	4.555	4.296	0.285	0.019	Xylanase proteins
*Bcpgx1*	2.536	1.212	0.496	−0.823	Polygalacturose proteins
*BCIN_02g04800*	4.861	4.796	0.611	0.536	Amino acid metabolism proteins
*BCIN_01g01440*	2.811	−1.449	2.354	−1.871	Dioxygenase TDA family
*BCIN_12g04510*	1.087	0.736	0.193	−0.165	Histidine kinase activity proteins
*BCIN_01g03510*	4.531	3.321	−2.06396	0.7321	Cytochrome P450
*BCIN_02g08880*	9.495	8.718	0.024	−0.752	Unknown function hypothetical proteins
*BCIN_02g08890*	9.104	8.745	0.565	0	Unknown function hypothetical proteins

## Supplementary Materials

The following are available online at https://www.mdpi.com/1422-0067/21/14/4865/s1.
