# Supplementary material for: Cytological and Gene Profile Expression Analysis Reveals Modification in Metabolic Pathways and Catalytic Activities Induce Resistance in Botrytis cinerea Against Iprodione Isolated From Tomato
Source: Int J Mol Sci. 2020 Jul 9;21(14):4865. doi: 10.3390/ijms21144865 (PMC7402349; doi:10.3390/ijms21144865)
Supplement: Supplementary file 1 [file ijms-21-04865-s001.zip › supplementary/supplementary data/supplementary.pdf]

International Journal of
Molecular Sciences

Table 1. 112 isolates of Botrytis cinerea collected from tomato plants and fruits in different locations of Guangxi province, China.

Location	Number of Isolates
Bichen lake	25
Hope town	23
Agriculture science park	14
Baiyu town	24
Dongwang village	6
Toutang town	6
Baise city	14
**Total**	**112**

Table 2. Mutants growth rate on drug free PDA medium with different generations.

Mutant	Mycelial Growth rate (%)			
	First Generation	Fifth Generation	Tenth Generation	Fifteenth Generation
**W0(B67)**	45.41±0.8a	48.76±1.43a	46.32±2.21a	49.77±2.27a
**M0**	47.01±0.6a	44.04±3.65a	48.99±1.21a	48.80±2.50a
**M2**	50.86±3.31a	51.65±1.1a	52.13±1.07a	51.86±1.89a

Note: The growth rate was observed after 2 days of each generation. B67 strain considered as wild type (W0).

Table 3. List of standard solutions of proteins.

	1	2	3	4	5	6
**Standard proteins**	0.00	0.01	0.02	0.03	0.04	0.05
**Distilled water**	0.10	0.09	0.08	0.07	0.06	0.05
**Coomassie brilliant blue**	5.00	5.00	5.00	5.00	5.00	5.00

Table 4. List of standard dilution series.

No. of samples	Concentrations	Dilution
**Standard**	64mU/L	
**5**	32mU/L	150μl of standard + 150μl dilution
**4**	16mU/L	150μl of standard No.5 + 150μl dilution
**3**	8mU/L	150μl standard No.4 + 150μl dilution
**2**	4mU/L	150μl standard No.3 + 150μl dilution
**1**	2mU/L	150μl standard No.2 + 150μl dilution

a) Add 50μl of each sample is a microplate (40μl of the product dilution and 10μl of tested sample)

b) Control wells without samples (rest of the procedures is same).

c) The absorbance (OD value of each well is measured in sequence with blank reading and at 450nm.

d) Concentration and OD value of the standards used to calculate the linear regression equation of the standard curve.

e) Each treatment has three replicates

f) The standard curve measured by the Coomassie brilliant blue method is $y=-21.55x^2+154.66x-7.9556$ $R^2=0.9984$.

Table 5. List of q-RTPCR Primers.

Genes name	Primers	Sequence 5'–3'
BCIN_13g03170	WHY-1-F	ACTCTTCCGCAGACACGC
	WHY-1-R	GCATCACAGCCAAACATAG
bcoah	WHY-2-F	GCTTGGCTACGACGAGTG
	WHY-2-R	TGCTTCCTTGGTGGTGAT
BCIN_08g01800	WHY-3-F	TGGTGCTGCTATGAACGC
	WHR-3-R	AAGCCAAAGGGTCGGAAG
BCIN_12g01530	WHY-4-F	TGGAGTCAAACGGGAAAT
	WHY-4-R	GGCCGTCAGACTAGCATC
BCIN_04g01200	WHY-5-F	GCGTGTACGAGTGGCTGAT
	WHY-5-R	CCATTCCCGTCCTCCTTT
Bccyp51	WHY-6-F	TGGTGCTGGCAGACATAG
	WHY-6-R	GAATAAACTTGCGTAATCG
BCIN_12g03660	WHY-8-F	AGGCTTCATGGTGTTGTG
	WHY-8-R	GAGGAAGGAGCACCGAAT
UBQ	WHY-UBQ-F	AGTAGCCAGCAGGTCAGA
	WHY-UBQ-F	TATTTCGCATCAATCCAG

Table 6. List of five highly sensitive isolates of *B. cinerea* to iprodione.

	**Inhibitory Rate (%)**				
**Conc. µg/mL**	**B311**	**B67**	**B614**	**B82**	**B111**
**0.01**	11.78	11.92	14.57	12.34	13.87
**0.02**	25.98	14.84	21.56	34.65	19.64
**0.04**	30.82	25.79	32.16	46.72	23.98
**0.1**	54.68	30.90	61.56	58.72	41.58
**1**	69.49	73.97	66.83	76.12	74.74
**2**	90.03	93.40	86.18	94.49	88.72

Table 7. Regression and correlation analysis of five higher sensitives isolates of *B. cinerea* to iprodione.

Isolate	Toxicity regression equation	Correlation coefficient (R^2)	EC50 (µg/mL)
**B311**	y=5.8283+0.9300x	0.9723	0.1286
**B67**	y=5.8699+1.1110x	0.9781	0.1648
**B614**	y=5.7342+0.8439x	0.9574	0.1349
**B82**	y=6.0889+0.9752x	0.9597	0.0765
**B111**	y=5.7717+0.9731x	0.9942	0.1610
**Average**			**0.1332**

Table 8. List of five moderate sensitive isolates to iprodione.

	Inhibitory Rate (%)				
**Conc. μg/mL**	**B215**	**B72**	**B76**	**B113**	**B115**
**0.02**	7.65	9.71	8.55	10.79	6.21
**0.05**	25.68	19.90	24.19	13.99	10.56
**0.1**	48.63	28.88	27.73	30.03	15.84
**1**	74.04	61.89	63.72	49.27	45.96
**2**	84.43	78.88	87.32	52.77	51.86
**3**	91.26	81.88	92.04	74.34	72.67
**5**	92.90	94.90	94.10	93.88	89.13

Table 9. Regression and correlation analysis of five moderate sensitives isolates to iprodione.

Isolate	Toxicity Regression Equation	Correlation Coefficient (R^2)	EC50 (μg/mL)
**B215**	y=5.7422+1.1217x	0.9846	0.2179
**B72**	y=5.5316+1.10994x	0.9856	0.3285
**B76**	y=5.6910+1.2.25x	0.9887	0.2663
**B113**	y=5.2597+0.9553x	0.9329	0.5348
**B115**	y=5.0871+01.0371x	0.9708	0.8243
**Average**			0.4334

Table 10. Protein concentrations of mutant and its wild type.

Isolates	Absorbance I	Proteins Concentration μg/mL	Absorbance II	Proteins Concentration μg/mL	Average
**Wild type (W0)**	0.79	1008.97	0.81	1038.97	1023.97a
**Mutant (M0)**	0.72	933.390	0.74	949.37	941.38b
**Mutant (M1)**	0.70	901.20	0.71	914.85	908.02bc

Table 11. Enzymatic activity of mutant and its wild type.

Enzymes	CE (U/g)	PG (U/g)	PMG (U/g)
**Wild type (W0)**	27.48±0.51a	214.07±1.46b	418.10±0.13b
**Mutant (M0)**	34.98±1.34b	277.36±7.40a	468.22±2.06a
**Mutant (M1)**	32.88±1.14b	196.07±4.76Ac	366.84±1.21c

Table 12. List of Class codes.

Class code	Description	Number
=	Complete match of intron chain	13639
c	Contained	3
e	Single exon transfrag overlapping a reference exon and at least 10 bp of a reference intron, indiction a possible pre-mRNA fragment	214
j	Potentially novel isoform (fragment):at least one splice junction is shared with a reference transcript	3574
o	Generic exonic overlap with a referfence transcript	372
p	Possible polymerase run-on fragment (within 2Kbases of a reference transcript	386
s	An intron of the transfrag overlaps a reference intron on the opposite strand (likely due to read mapping errors	46
u	Unknown, intergenic transcript	1229
x	Exonic overlap with reference on the opposite strand	858
i	Transfrag falling entirely within a reference intron	0
r	Repeat. Currently determined by looking at the soft-masked reference sequence and applied to transcripts where at least 50% of the bases are lower case	0
.	Tracking file only, indicates multiple classifications	0

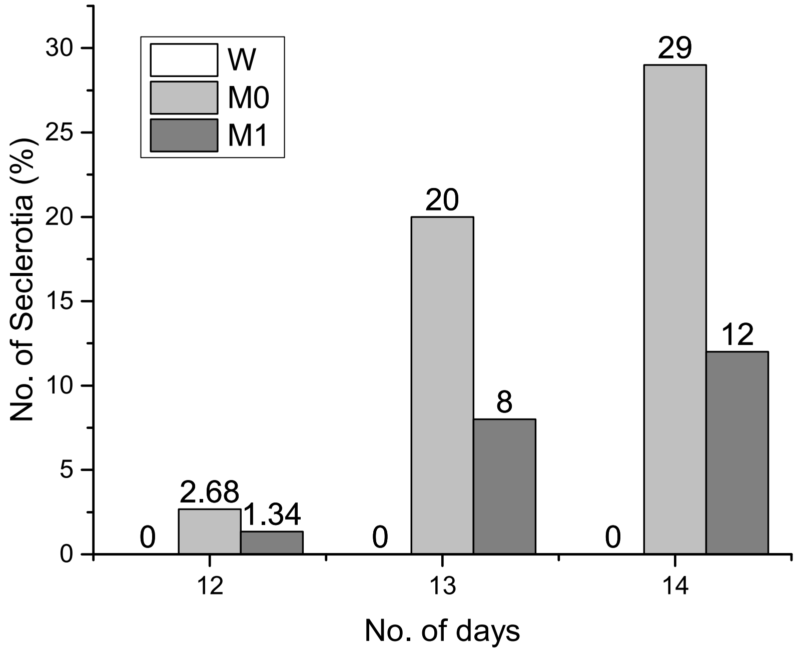

Figure S1. Sclerortia formation after 12, 13 and 14 days of mutants and its wild type on PDA media amended with 5μg/mL.

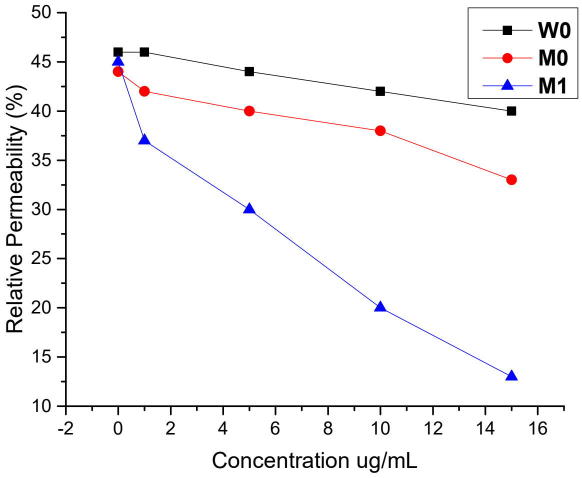

Figure S2. Relative permeability of wild type and its mutants with different concentrations of iprodione (μg/mL).

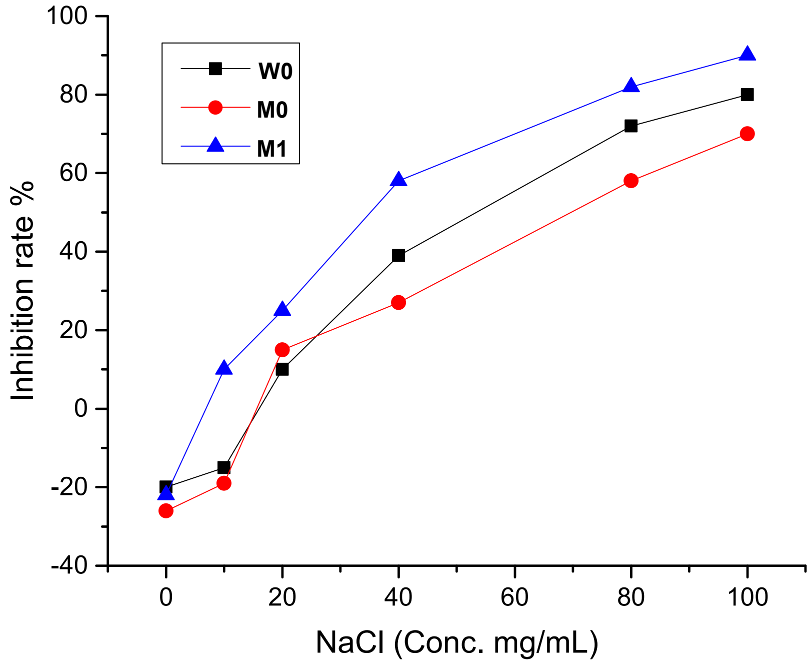

Figure 3. Osmotic sensitivity of wild type and its mutants with different NaCl (mg/mL) concentrations.

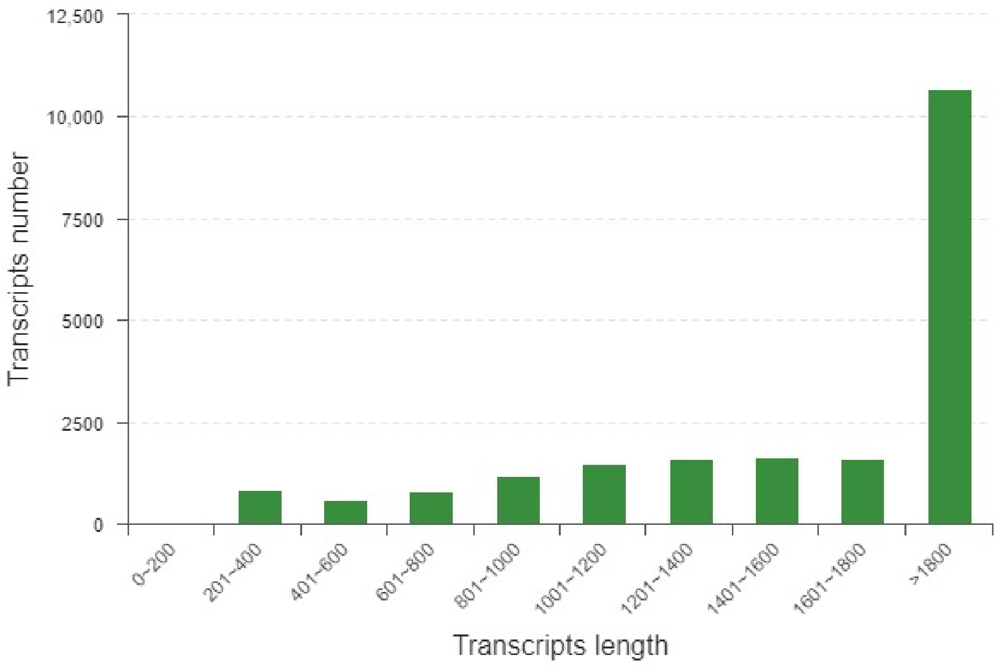

Figure 4. Length distribution of transcripts.

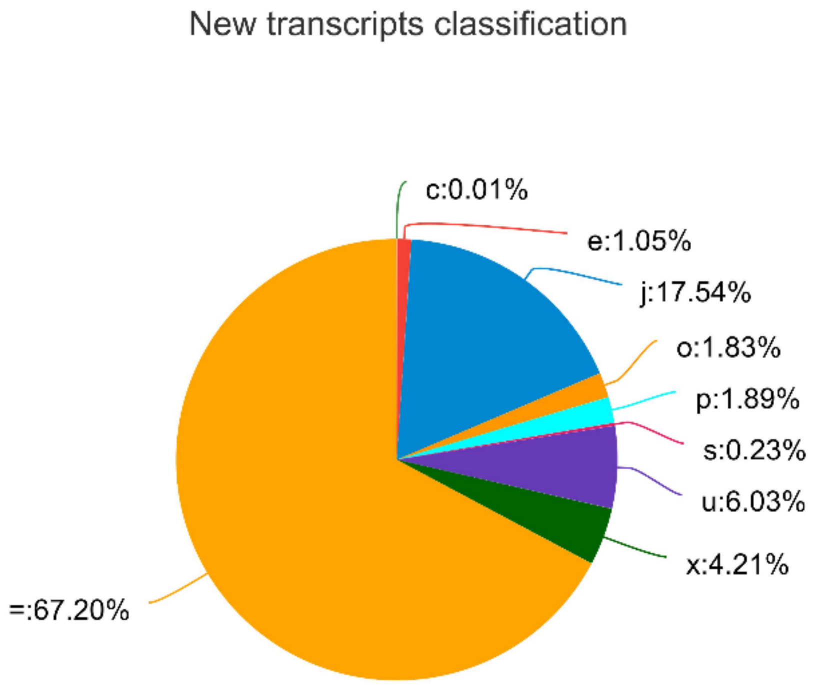

Figure S5. Classification of new transcript in RNA sequencing data

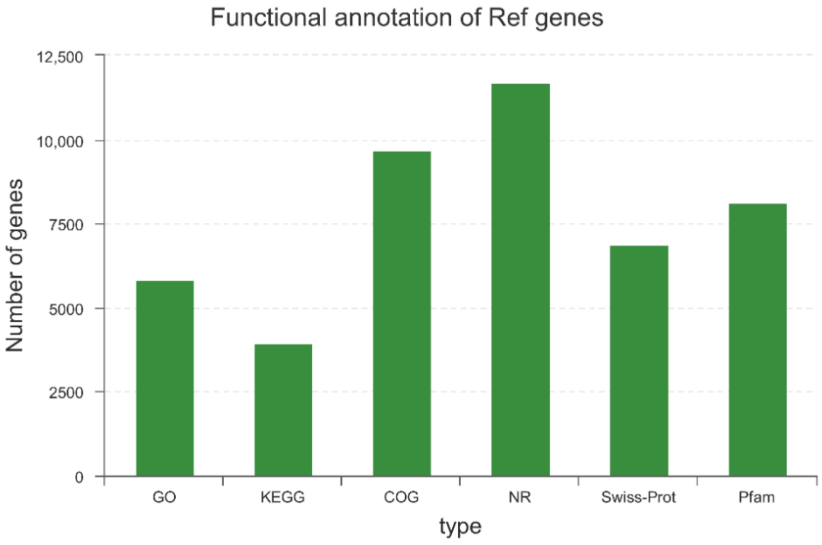

Figure S6. Annotation of unigenes genes into six databases (GO, KEGG, COG, NR, Swiss-Prot and Pfam) on the bases of their function.

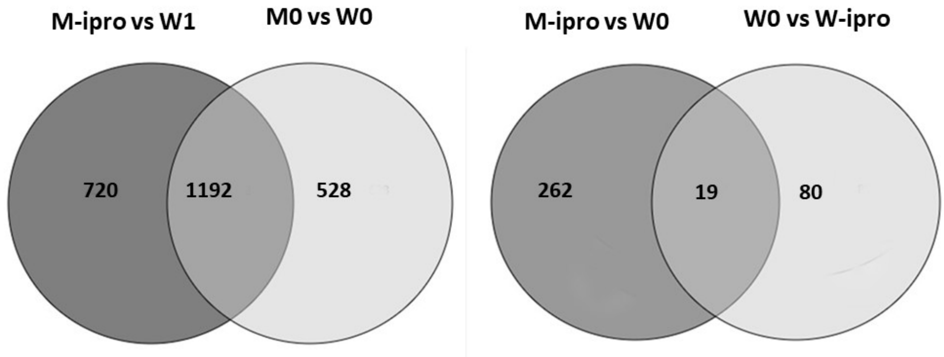

Figure S7. The Venn diagram represent the number of DEG's in wild type (W0, W1) and its corresponding mutant (M0 vs Mipro) with or without iprodione treatment. **(a)** M-ipro vs W-ipro and M0 vs W0 showed (720, 528) unique DEG's respectively and 1192 common DEG's; **(b)** Mutant (M1 vs M0) and wild type (W1 vs W0) with or without iprodione treatment shared only 19 common DEG's and (262, 80) unique DEG's respectively. The cut off limit of DEG's was less than (FDR=0.05) and greater than log fold 2 change value.

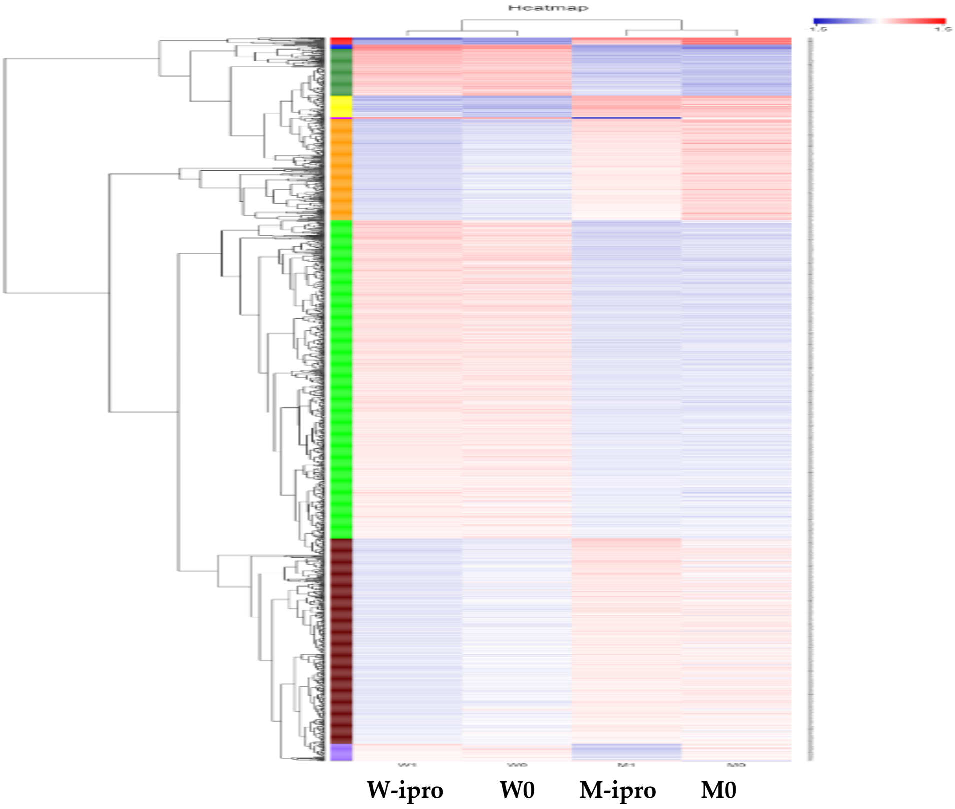

Figure S8. Herrirachial Clusture analysis of mutant and wild type with and without iprodione treatment.
